# Utilization of Posterior Column Osteotomies Prior to Optical-Guided Navigation in Instrumented Lumbar Spine Surgery

**DOI:** 10.7759/cureus.97477

**Published:** 2025-11-21

**Authors:** Alexander J Schupper, Jeremy Steinberger, James Lin

**Affiliations:** 1 Neurosurgery, Icahn School of Medicine at Mount Sinai, New York, USA; 2 Neurological Surgery, Mount Sinai Health System, New York, USA; 3 Orthopedic Surgery, Mount Sinai Medical Center, New York, USA

**Keywords:** 3d surgical navigation, adult idiopathic scoliosis, lumbar instrumentation, scoliosis, spine surgery, thoracic instrumentation

## Abstract

Optical topographic imaging (OTI)-guided navigation allows for accurate navigation even after posterior column osteotomies (PCOs), giving spine surgeons the option to place instrumentation after PCOs. Preoperative computed tomography (CT) imaging can be used safely and effectively for optical-guided navigation in cases where PCOs are performed.

A middle-aged woman presented with symptomatic adult idiopathic scoliosis (AdIS). After unsuccessful conservative treatment, she underwent spinal deformity correction with posterior instrumented fusion using OTI-guided navigation. During surgery, PCOs were performed at the apical levels before instrumentation placement. After the osteotomies, accurate OTI registration was achieved using the remaining laminar surfaces, and pedicle screws were placed under navigation guidance. The patient made a full recovery, with resolution of her back and radicular pain.

## Introduction

The accuracy of pedicle screw instrumentation in spine surgery has evolved from a goal to an accepted standard [1]. With the increasing availability of computer-assisted navigation and intraoperative computed tomography (CT), surgeons now have more tools to confirm proper instrumentation placement before leaving the operating room [2]. Optical topographic imaging (OTI) is a novel navigation technique that relies on surface osseous anatomy for registration, and preservation of bony anatomy is generally recommended to maintain registration accuracy.

Traditionally, in deformity surgery, bony releases through osteotomies are performed before screw placement to improve spinal flexibility and to provide anatomical landmarks for “freehand” pedicle screw insertion. In addition, once pedicle screws are placed, performing osteotomies, such as partial or complete facet removal, becomes more difficult. However, the introduction of intraoperative navigation has shifted this workflow, as these systems depend on bony landmarks to verify accuracy. Optical-guided navigation in particular requires visualization of these landmarks to register to a preoperative CT image. For this reason, performing osteotomies before optical-guided navigation registration is not routinely done and has not been previously reported in the literature.

In this presentation, we demonstrate accurate optical-guided navigation registration for pedicle screw placement following posterior column osteotomies (PCOs).

## Case presentation

A 46-year-old woman with adult idiopathic scoliosis (AdIS) presented with several years of worsening back pain on the left side, along the convexity of her lumbar curve, and right leg radiculopathy radiating into the groin and anterior thigh. She had not improved with extensive physical therapy, epidural steroid injections, chiropractic treatment, or anti-inflammatory and muscle relaxant medications. She was also dissatisfied with her cosmetic appearance due to a left-sided truncal shift and a right-sided asymmetric waist crease.

On physical examination, she had 5/5 strength in all major muscle groups except for 4/5 strength in the right hip flexor and tibialis anterior. Sensation was intact in all dermatomes, and no long-tract signs were present.

**Figure 1:**
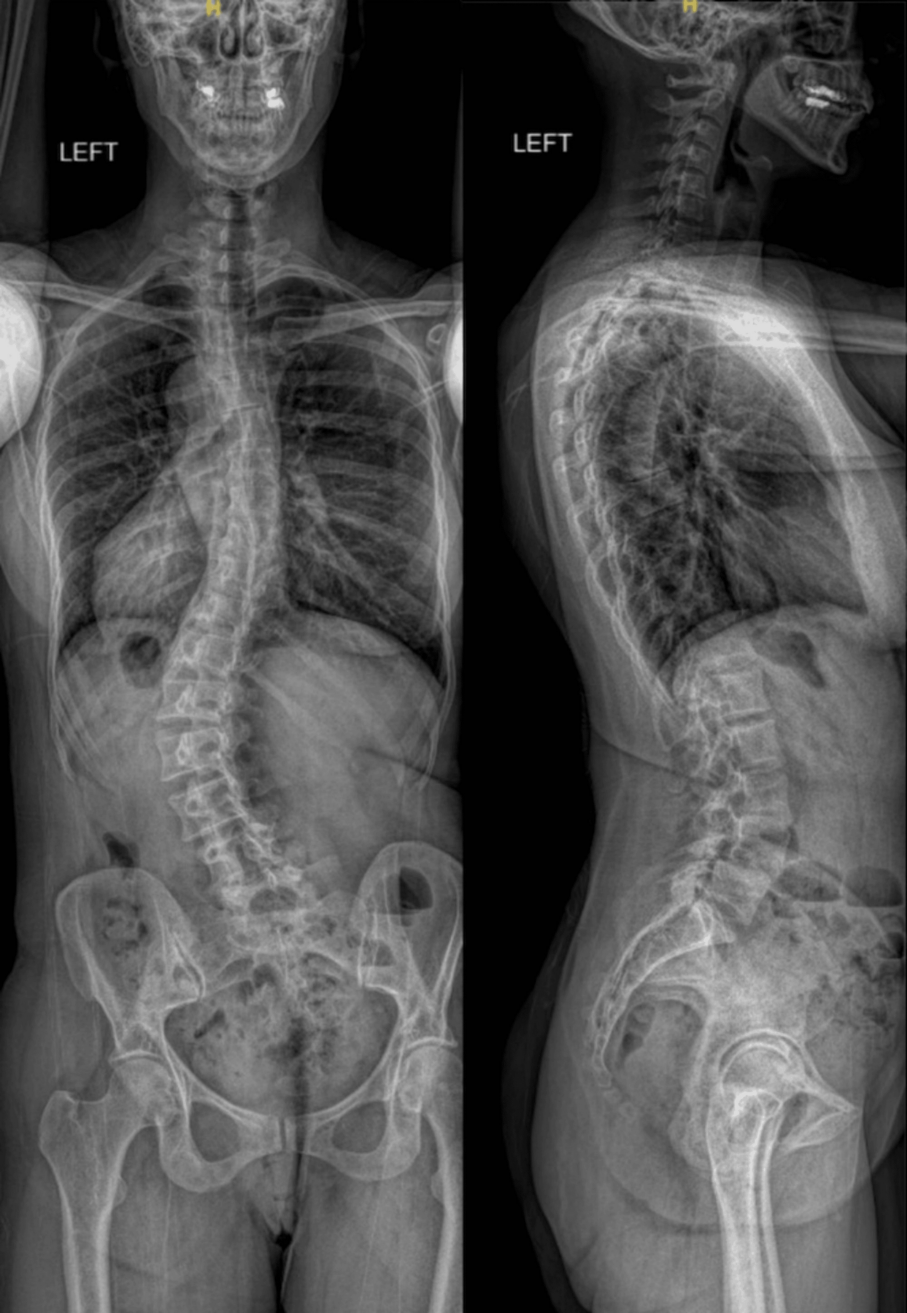
Preoperative anteroposterior (left) and lateral (right) long-cassette radiographs demonstrating levoscoliosis with a thoracolumbar curve apex at the L2 and associated coronal malalignment.

**Figure 2:**
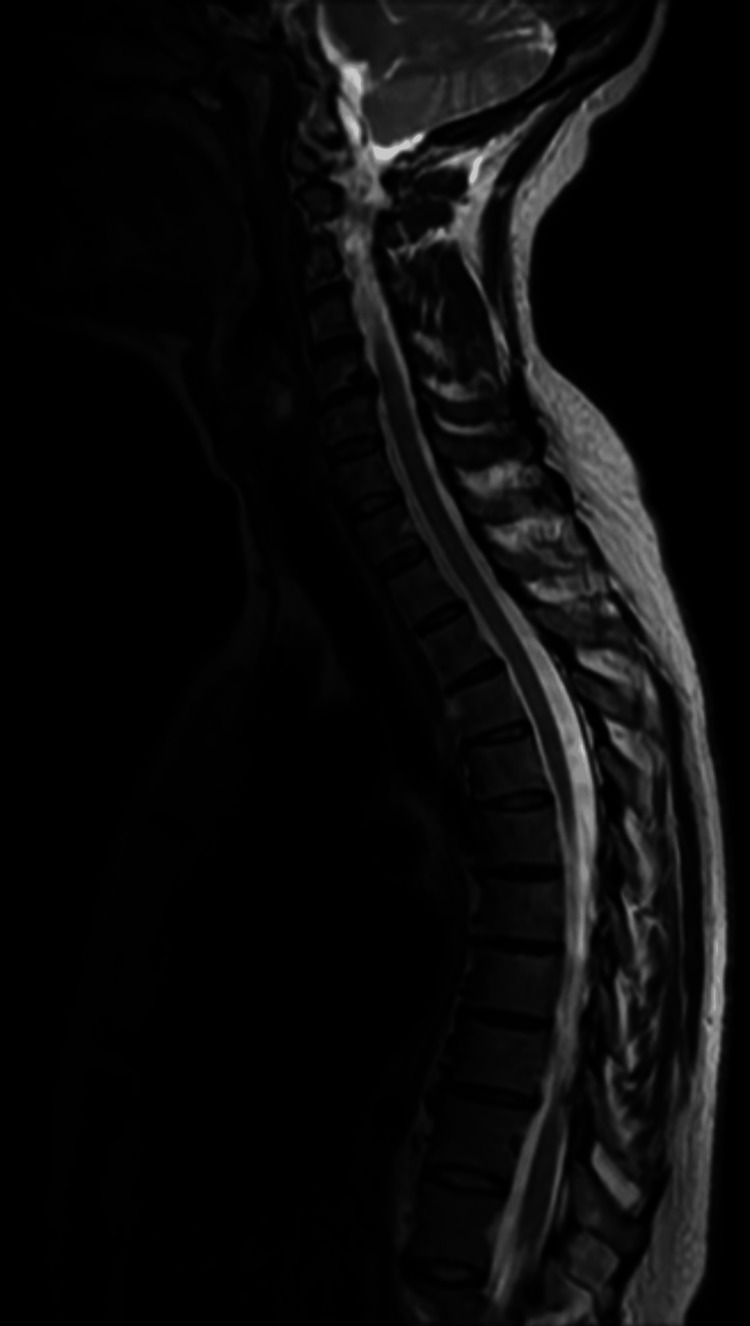
Preoperative sagittal T2-weighted MRI.

Preoperative imaging demonstrated a 45° lumbar curve with the apex to the left, a 17° lumbosacral fractional curve with the apex to the right, −4 cm coronal imbalance, and preserved sagittal alignment (Figure 1). According to the AdIS classification, this was categorized as a 5/NS/Cor malaligned curve [3]. Magnetic resonance imaging (MRI) showed no focal stenosis, cord tethering, or syrinx (Figure 2).

The patient was indicated for T10-ilium posterior instrumented fixation with PCOs (Schwab Grade 2) at L2-L4, performed under neuromonitoring. Intraoperative optical-guided navigation was used based on a preoperative CT scan with the 7D (SeaSpine) navigation system.

Surgical technique

**Figure 3:**
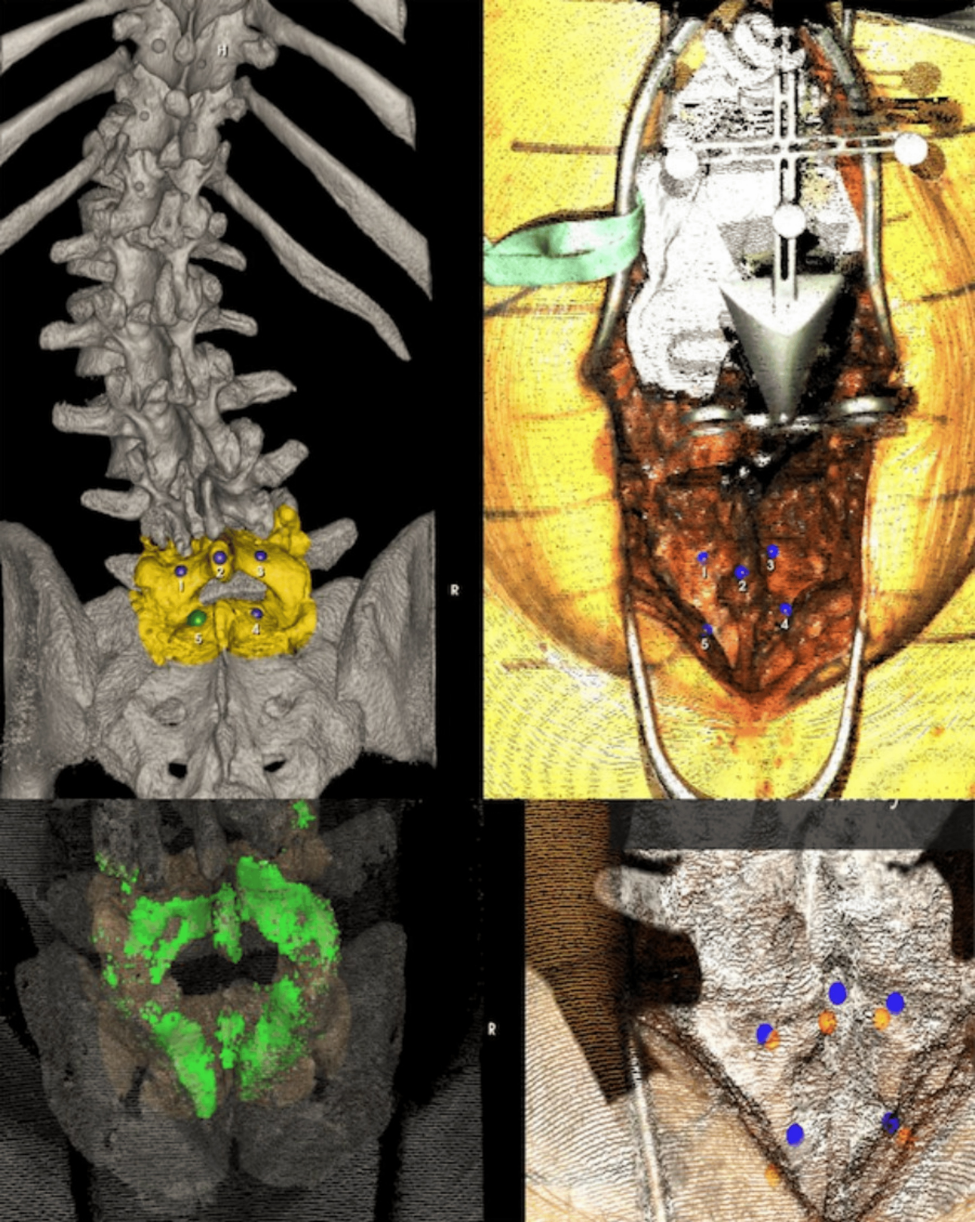
Intraoperative imaging showing the preselected fiducial points on the preoperative CT and in situ (top) at the L2-L3, along with post-registration FLASH points confirming accurate registration. Posterior column osteotomies have been performed at these levels.

**Figure 4:**
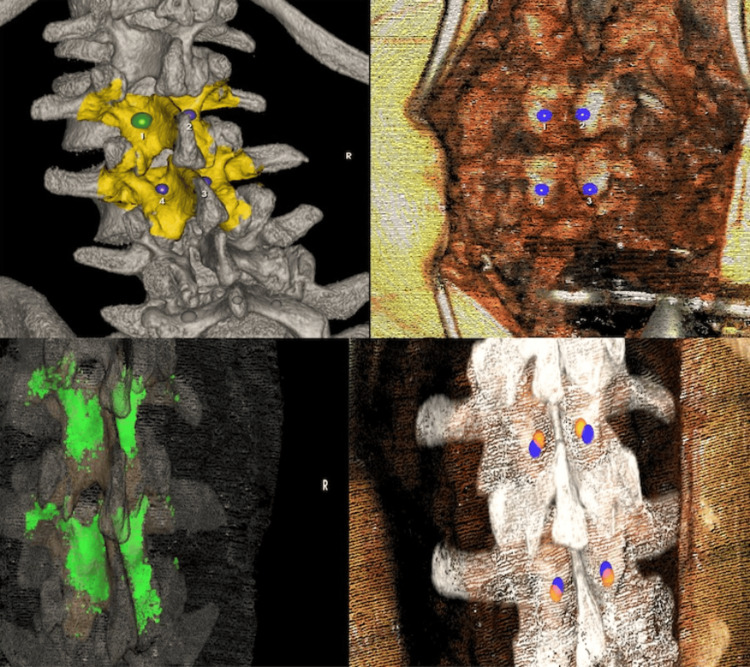
Intraoperative imaging showing the preselected fiducial points on the preoperative CT and in situ (top) at the L5-S1, along with post-registration FLASH points confirming accurate registration.

Following intubation and neuromonitoring electrode placement, the patient was positioned prone on a Jackson table. The spine was exposed using subperiosteal dissection from the T10 to the pelvis. Inferior facetectomies were performed from the T11 to L5. The navigation array was then placed on the L4 spinous process, and five registration points were selected on the L5 spinous process and lamina, as well as the S1 lamina (Figure 3). After a 5.4-second FLASH image, 5,045 points were matched to the preoperative CT scan, and accuracy was confirmed (Figure 3). Pedicle screws were placed from L4 to S1, and bilateral S2-alar-iliac (S2AI) screws were inserted. PCOs (Schwab Grade 2) were then performed at the apical levels (L2-L4). This involved removing the spinous process, developing the midline raphe between the ligamentum flavum leaflets, and using 3-mm Kerrison rongeurs to remove the ligamentum flavum and superior articular process bilaterally. Additional FLASH images were obtained to separately register the L2-L3 and T10-L1 levels (Figure 4). After screw placement, the lumbar apex was de-rotated, and pre-contoured rods were placed to achieve correction. The patient recovered well and was discharged home on postoperative day (POD) 4 without neurological deficits.

Results

**Figure 5:**
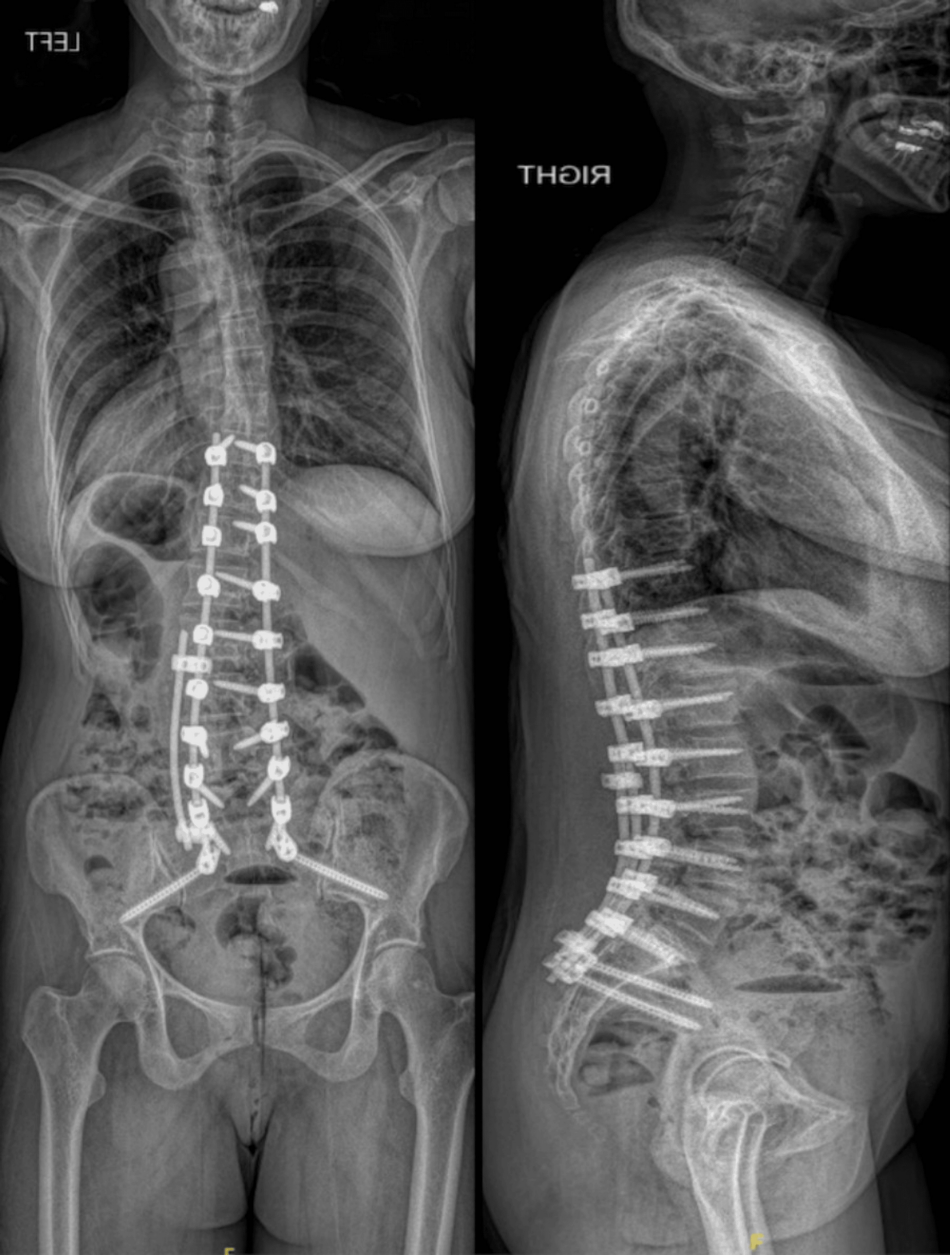
Postoperative anteroposterior (left) and lateral (right) long-cassette radiographs demonstrating T10-ilium posterior instrumentation, correction of the preoperative coronal deformity, and maintenance of global sagittal alignment.

The patient recovered well after surgery and was discharged home on POD4. Postoperative scoliosis radiographs show correction of her lumbar curve and coronal malalignment, with preservation of sagittal alignment through appropriate placement of the T10-ilium instrumentation (Figure 5).

**Figure 6:**
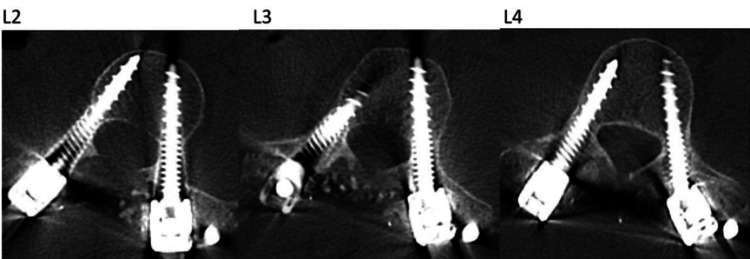
Postoperative CT imaging demonstrating accurate pedicle screw instrumentation at the L2 through L4 apical levels.

Axial CT images show accurate screw placement at the osteotomy levels (L2-L4) across the apex of her scoliosis (Figure 6).

At her most recent follow-up, the patient was doing well, with resolution of her prior symptoms and no residual pain, and she was no longer taking pain medication.

## Discussion

Accurate pedicle screw instrumentation is a critical component of modern spine surgery. The use of navigation guidance has been shown to improve the accuracy of pedicle screw placement [1,2,4]. Recently, OTI-based navigation has become available, using topographic bony landmarks merged with a preoperative CT scan, eliminating the need for an intraoperative CT. Preoperative CT offers advantages over intraoperative CT, including decreased surgical time and reduced radiation exposure to both surgical and anesthesiology staff [5]. However, with OTI navigation, preservation of bony anatomy is typically recommended to maintain registration accuracy. The purpose of this study is to illustrate that accurate OTI registration can still be achieved after PCOs, an application not previously described.

In this case, OTI-based navigation (7D, SeaSpine) was used to guide pedicle screw placement in a patient with AdIS undergoing T10-pelvis fusion. Registration was successfully completed in the proximal lumbar spine even after PCOs were performed across the apical segments (L2-L4). Registration points were obtained from the remaining lamina, pars, and transverse process, and screws were placed under navigation guidance. The patient was discharged on POD4 with correction of her deformity.

OTI is a novel navigation technology that uses surface osseous anatomy to register to a preoperative CT scan. Unlike other navigation systems, OTI uses nonionizing structured light to generate a 3D surface map that aligns with the high-resolution CT, effectively providing thousands of fiducial points for registration and enabling a high degree of accuracy [6]. In an ex vivo feasibility study, Jakubovic et al. demonstrated that OTI offers strong accuracy in both cranial and spinal navigations, faster acquisition times, and improvements in workflow efficiency [7]. Stewart also reported on 150 consecutive spinal instrumentation cases using OTI navigation with no complications or screw malposition [8].

In deformity surgery, the intraoperative workflow varies by surgeon preference and training. Some surgeons perform all bony work, such as decompressions and PCOs, before placing instrumentation, while others prefer to instrument first. When using intraoperative CT-based navigation, either workflow is feasible. However, with OTI-based navigation, it has traditionally been recommended to preserve as much bony anatomy as possible to support accurate surface registration. The results of this study suggest that OTI-based navigation may be compatible with a “PCO-first” workflow, which may be preferred by many deformity surgeons.

To the authors’ knowledge, this is the first reported case of AdIS correction using optical-guided navigation in which accurate registration was demonstrated after osteotomies were performed. This finding is important, as it suggests that surgeons using similar technology do not need to alter their preferred workflow or risk increased operative time, additional radiation exposure, or higher rates of screw malposition. While this report is limited by the single-case design, larger case series will help further validate this workflow and highlight the benefits of accurate screw placement with optical-guided navigation without added time or radiation during scoliosis correction.

## Conclusions

Optical-guided navigation can be used after PCOs, allowing surgeons to maintain a traditional workflow of performing osteotomies before hardware placement while still benefiting from intraoperative navigation. This finding offers greater flexibility in deformity surgery workflow and may help facilitate instrumentation placement. Larger studies involving patients undergoing deformity procedures with osteotomies are needed to further evaluate the role of optical topographic imaging in spinal instrumentation navigation.
